# A Neural Network Approach to Reducing the Costs of Parameter-Setting in the Production of Polyethylene Oxide Nanofibers

**DOI:** 10.3390/mi14071410

**Published:** 2023-07-12

**Authors:** Daniel Solis-Rios, Luis Jesús Villarreal-Gómez, Clara Eugenia Goyes, Faruk Fonthal Rico, José Manuel Cornejo-Bravo, María Berenice Fong-Mata, Jorge Mario Calderón Arenas, Harold Alberto Martínez Rincón, David Abdel Mejía-Medina

**Affiliations:** 1 Grupo de Investigación en Ingeniería Biomédica, Universidad Autónoma de Occidente, Cali 760030, Colombia; daniel.solis@uao.edu.co (D.S.-R.); ceglopez@gmail.com (C.E.G.); ffonthal@uao.edu.co (F.F.R.); jorge_mario.calderon@uao.edu.co (J.M.C.A.); harold_alb.martinez@uao.edu.co (H.A.M.R.); 2 Facultad de Ciencias de la Ingeniería y Tecnología, Universidad Autónoma de Baja California, Tijuana 21500, Baja California, Mexico; luis.villarreal@uabc.edu.mx (L.J.V.-G.); bfong@uabc.edu.mx (M.B.F.-M.); 3 Facultad de Ciencias Químicas e Ingeniería, Universidad Autónoma de Baja California, Tijuana 21500, Baja California, Mexico; jmcornejo@uabc.edu.mx

**Keywords:** artificial neural networks, PEO nanofibers, electrospinning

## Abstract

Nanofibers, which are formed by the electrospinning process, are used in a variety of applications. For this purpose, a specific diameter suited for each application is required, which is achieved by varying a set of parameters. This parameter adjustment process is empirical and works by trial and error, causing high input costs and wasting time and financial resources. In this work, an artificial neural network model is presented to predict the diameter of polyethylene nanofibers, based on the adjustment of 15 parameters. The model was trained from 105 records from data obtained from the literature and was then validated with nine nanofibers that were obtained and measured in the laboratory. The average error between the actual results was 2.29%. This result differs from those taken in an evaluation of the dataset. Therefore, the importance of increasing the dataset and the validation using independent data is highlighted.

## 1. Introduction

Electrospinning is a technique used to produce polymeric fibers with myriad applications in various areas, such as tissue engineering [1], food preservation [2], water treatment [3], and drug distribution [4], among others. Notably, PEO electro-spun fibers have been reported to possess unique characteristics that make them ideal for use in several applications, such as being viscoelastic, water-soluble, biodegradable, biocompatible, and nonionic, with interesting mechanical properties, and so on [5]. Thus, various applications can use electro-spun fiber systems that are loaded with different molecules. These applications are detailed as follows:

- Topical drug delivery. According to the authors of [6], an average fiber diameter of ~1500 nm is needed, which could be produced using a polyethylene oxide/ethyl cellulose fiber loaded with an ibuprofen molecule.
- Connective tissue regeneration. According to the authors of [7], an average fiber diameter of ~8000–10,000 nm is needed, which could be produced using a polycaprolactone/polyethylene oxide loaded with a fibroblast growth factor (FGF-2) molecule.
- Food monitoring. According to the authors of [8], an average fiber diameter of ~3000 nm is needed, which could be produced using a polycaprolactone/polyethylene oxide loaded with a *Hibiscus rosa sinensis* extract molecule.
- Cartilage tissue repair. According to the authors of [8], an average fiber diameter of ~457 nm is needed, which could be produced using chitosan/polyethylene oxide.
- Supercapacitor applications. According to the authors of [9], a ~3500 nm average fiber diameter is observed, which could be produced using a polyaniline/polyethylene oxide loaded with an *m*-cresol molecule.
- Oral drug delivery system. According to the authors of [10], a ~1500 nm average fiber diameter is desired, which could be produced using a polymethyl methacrylate/polyethylene oxide loaded with a doxycycline molecule.

The production of nanomaterials (nanofibers) via electrospinning is affected by many operational parameters used in a general description of process-specific electrospinning, such as molecular weight (Da), solvent polymer solution concentration (%), viscosity (N·s/m^2^), syringe caliber (mm), voltage (kV), solution, collector–injector distance (cm), injector velocity (flow rate (mL/h), collector type, collector velocity (rpm), relative humidity (%), and temperature €. In this way, the average fiber diameter is an essential outcome parameter for functionalized electro-spun fibers [11]. For specific applications, fiber diameter will determine the mechanical properties, wherein reduced fiber diameters enhance the tensile properties, due to the improvement of crystallinity and molecular orientation [12]. Moreover, their thermal behavior and conductivity are also altered [13].

Thus, this technique must be optimized according to the specific purpose for which the fibers are intended, by predicting the average size of the fibers produced using the main parameters (Table 1).

The values of these parameters could affect nanofiber fabrication and nanofiber applications in various areas. Thus, the production of nanomaterials (nanofibers) via electrospinning is affected by many of the operating parameters that are not controlled [15] because there is no clear (linear) relationship between these variables and the outcome of the process.

**Figure 1:**
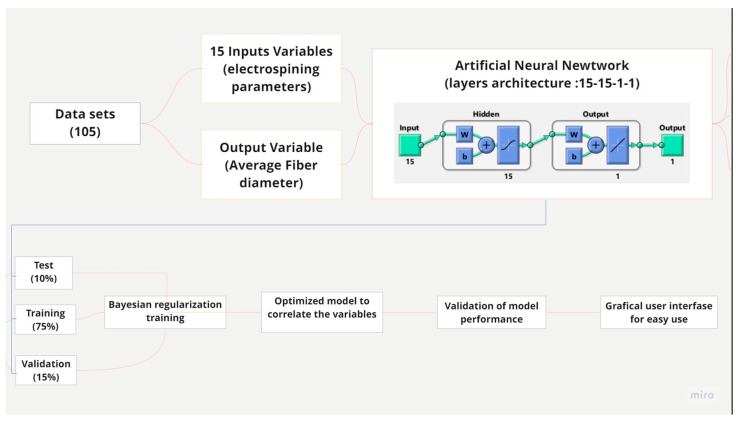
Schematic presentation of the ANN model development.

For this reason, an essential feature of ANNs has relevance because they do not require specific equation formats. This means that they only need enough meaningful input-output data to obtain the best mathematical model for prediction. A vital role is played by the training algorithm (Figure 1) because it holds the key to successfully predicting the process’s outcome. In this way, Bayesian regularized artificial neural networks (BRANN) are more robust than standard backpropagation networks and can reduce or eliminate the need for lengthy cross-validation because Bayesian regularization (training method) is a mathematical process that converts a nonlinear relationship, in the manner of ridge regression. This is an extension of linear regression, in which the loss function is modified to minimize the complexity of the model [16].

Neural networks have already been previously used for predicting the diameter of a nanofiber. Citing a few examples, in one study [17], a model was trained with a dataset of 18 nanofibers, with solution concentration, conductivity, electric field strength, and flow rate as the input parameters. The results show a correlation of 0.9992 between the real and the predicted datasets. Brook and Tucker [18] also present a predictive model using 13 input parameters. The results are delivered via the relative percentage error, with a value of 22.3%.

Additionally, Ketabchi et al. [19] present a model for predicting nanofibers with chitosan/polyethylene oxide (PEO). The model has four input variables and was trained with a 40-element dataset. It was validated with ten elements and presented results with an MSE of 0.0707 and a correlation of 0.9630.

## 2. Materials and Methods

### 2.1. Methodology

In performing this analysis, a methodology was followed with three main stages:

1. Creation of the dataset for training the intelligent prediction model.
2. Training and optimization of the intelligent prediction model.
3. Validation of the intelligent prediction model.

Each stage is detailed in the following sections.

### 2.2. Creation of the Dataset for Training the Intelligent Prediction Model

The dataset used to train the ANN model was gathered from experimental results reported in the literature [20,21,22,23,24,25,26,27,28,29,30,31,32,33,34,35,36,37] (see the Supplementary Materials for details; Table S1: Full precision ANN weights; Video S1: The model fitting process.), and references more than 100 experiments. Since the dataset was taken directly from the literature, it was necessary to make some adjustments to standardize all the data before using it to train the neural network.

This search was performed in different recognized scientific databases such as Science Direct, Springer, and Google Academic, among others, using filters encompassing at least five years from when the research began in 2021, meaning that the earliest papers were published in 2016. Additionally, the filter ‘“PEO” and “electrospinning”’ was applied.

Thus, once the information was collected, a matrix with the most relevant data for training the neural network was built. The method that was used to realize the matrix involves sourcing the experimental measurements of the parameters for electrospinning that were used for the dataset; at least 100 units of data are required to achieve good training of the neural network in the Bayesian regulation method [16]. The dataset includes: (a) reference data: the reference from which the data was extracted, the corresponding author’s country, and the published year; (b) parameters of the solution: molecular weight (Da), solvent, polymer solution concentration (%), and viscosity (N·s/m^2^); (c) electrospinning parameters: voltage (kV), collector–injector distance (cm), injector velocity (flow rate (mL/h)), collector type, collector velocity (rpm), and syringe caliber (mm); (d) ambient parameters: relative humidity (%) and temperature (°C); € fiber diameter results: average diameter of the fibers (nm), fiber standard deviation (nm), minimum fiber diameter (nm), and maximum fiber diameter (nm).

After collecting the data, we pre-processed the data as follows.

Verifying units and rounding of significant figures for the optimal neural network processing was performed, as well as coding equivalence to process the solvents in the artificial neural network, which were weighted according to their importance (Table 2).

A data normalization process was also carried out by distributing the values between 0 and 1. Data normalization is an essential pre-processing step that involves transforming features in a specific range so that the larger numeric feature values cannot dominate the smaller numeric feature values [25].

Furthermore, binary coding was also performed, where zero (0) or one (1) was assigned when there was a static collector or a rotary collector, respectively. This coding will depend on the injector velocity (flow rate (mL/h)), where, in the case of a stationary collector, there will be no velocity, giving this item a value of zero. In the opposite case, a one (1) is assigned, and the respective speed that has been processed during the test as reported in the literature is given. All this is used to be able to predict the average fiber size satisfactorily.

It is important to note that information about some of the input parameters in 105 records was unavailable. Table 3 shows the distribution of data by parameter.

Thus, to address the issue of missing data, we generated synthetic data; this can be valuable when real-world data are expensive, scarce, or simply unavailable [38]. Additionally, in an ANN, an abundance of data helps to stabilize the predicted results and optimize the start point. Synthetic data are used to feed the dataset and help the IA to learn [39]. Synthetic data, such as temperature and the percentage of humidity, are taken from natural conditions that can be achieved in a laboratory. Moreover, it is not recommended to leave blank spaces in a dataset where these parameters were obtained in a study because not all studies reported all critical parameters; ambient parameters affect the resulting fiber diameter [40]. It is important to note that variations in outdoor and indoor climate conditions are high between laboratory tests; definitive correlations and percentage errors for all laboratory conditions cannot be expected. Hence, the obtained results from this study will be approximate and will act as a guide to present a reference set of requirements to start with, after which each researcher will make the corresponding adjustment that their own laboratory needs require. Thus, we implemented a diverse group of strategies for data generation, which are detailed as follows:

Web search for the ambient parameters. The temperature and humidity in the laboratories are taken as environmental parameters. For this reason, in those cases where this information was not specified, a search was performed in specialized sources where there are historical records of the climate according to locality; thus, inquiries were made, using as parameters the location of the city where the university of the authors of each article is located, along with the approximate weather when the experiment was carried out, according to its publication date.

ANN-generated data. With the parameter of syringe caliber presenting 61 original records and viscosity presenting 48 original documents, a neural network with an 11-11-1-1 architecture was created, using the Levengber–Marquardt algorithm in the MATLAB computational tool for each parameter. To complete the missing data, most parameters were included except the environmental ones, and the collector speed, with the particularity that the average fiber diameter was an input, managing the division of the dataset with a split of (75-15-10) for training, validation, and testing. The activity results include an MSE of 5.58 for viscosity and syringe caliber, along with correlation indexes of 0.994 and 0.9504, respectively.

### 2.3. Training and Optimization of the Intelligent Prediction Model

The performance of a neural network is known to be highly sensitive to hyperparameter settings, and deep learning researchers often spend long hours trying to tune the hyperparameters. This means that the performance of the trained model is strongly related to the hyperparameter tuning process.

Among the most relevant parameters are the number of input variables (features), the number of hidden layers and neurons in each layer, the activation function used in each layer, the optimization algorithm used, the learning rate, epochs, batch size, and even the programming environment used.

Then, as a strategy to obtain a prediction model with a high degree of correlation to the results obtained during the physical process of nanofiber production, MATLAB 2019b (Natick, Lakeside Campus 1 Lakeside Campus Drive Natick, MA USA) and Python 3.9.11. (512 Lafayette Boulevard, Suite 2, Fredericksburg, VA, United States) were used as programming environments to train the nanofiber diameter prediction model. Thus, every stage of hyperparameter tuning was tested in both environments.

To obtain a prediction model, the 105 records in the dataset were used randomly, divided as follows:

Training. This is a process in which the weights and parameters of the prediction model are adjusted through machine learning algorithms. For this task, 75% of the data from the dataset was used.

Validation. At this stage, the resulting model is evaluated, with data that are not used in the training process, to determine the need to adjust the hyperparameters of the network, such as input and hidden layers, neurons, learning rate, epochs, the learning rate, and the size of the batch, among others. At this stage, 15% of the dataset was used.

Testing. Finally, once the model is fitted, it is retested with data that have not been tested previously. For this work, this stage was carried out in two ways: first, using the remaining 10% of the dataset, then, with a set of nine nanofibers that the authors of this work had produced.

**Figure 2:**
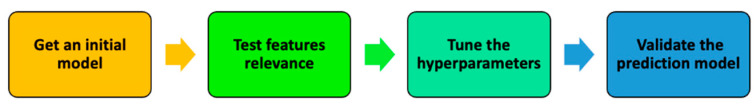
Schema of the hyperparameter tuning process.

The hyperparameter adjustment process comprised a series of different steps, as shown in Figure 2.

#### 2.3.1. Establishing an Initial Model

Once a base model was identified, the next stage began. The first step was to choose an initial network configuration that would generate a set of theoretical results with a correlation of at least 0.9 with the actual results. This process was carried out empirically, based on the knowledge and experience of the authors.

#### 2.3.2. Test Feature Relevance

The selection of input variables (features) directly influences the results obtained by the neural network. Therefore, it is crucial to perform this process correctly. Although there are various algorithms to select the appropriate features, in this work, the decision was made to use the 15 parameters identified in the literature since these matches the way that the physical process of nanofiber generation is carried out. Subsequently, the impact that each of the parameters has on the result of the prediction model.

The premise was that if a parameter contributed to decreasing the correlation of the resulting diameters with the real set, that parameter would not be included in the final model. Thus, seven initial parameters were selected empirically, then later, parameters were added to determine their impact on the final result. Table 4 shows the results of this exercise to evaluate the relevance of the input variables.

Thus, the results show that the 15 features are very relevant as input variables. Therefore, the next step is to adjust the hyperparameters.

Table 5 compares the obtained percentage error from the dataset with that of the real cases, considering the 15 identified parameters. In this comparative exercise, the parameters such as the solvent, the collector type, and the rotatory collector velocity are the parameters that showed a higher percentage error, these being parameters with high variability, provoking an increase in the fiber diameter’s standard deviation. Conversely, parameters such as the syringe’s caliber, voltage, flow rate, and collector–injector distance are parameters with less variance, provoking a lower probability of an increase in the potential percentage error in the final results; these last parameters are the most important for controlling the final fiber diameter [14].

#### 2.3.3. Tuning the Hyperparameters

The performance of a neural network is susceptible to hyperparameters. Therefore, modifications were made to optimize the result. Table 6 shows the adjustment process and the results obtained.

Then, the architecture of the model to be used was set at 15-15-1-1 since this is the format that offered the best performance for the prediction model.

#### 2.3.4. Validating the Prediction Model

The validation of the prediction model was carried out in two stages. The first stage consisted of using 15% of the dataset that had yet to be used to evaluate the correlation with the results of the prediction model. Subsequently, the model that obtained the best correlation was used and validated with nine nanofiber samples made in a laboratory. Section 2.4 elaborates on this process.

### 2.4. Nanofibers for the Validation Process

The following process used a set of nanofibers to validate the prediction model.

#### 2.4.1. Materials

Polyethylene oxide (PEO) (600,000 Da, Sigma Aldrich, St. Louis, MO, USA), Bradford reagent (1.4 mg/mL, Millipore, Burlington, MA, USA), and distilled water (Sigma Aldrich, St. Louis, MO, USA) were used as received.

#### 2.4.2. Preparation of Polyethylene Oxide PEO Solutions

The PEO solution made with distilled water was prepared in a percentage, as follows: 6.5% (0.65 g) of PEO and 93.5% (9.35 g) of H_2_O for a total of 100% (10 g). The solution was stirred for 30 min at room temperature to achieve homogeneity of viscosity.

#### 2.4.3. Parameters of the Electrospinning Technique

Solidwork^®^ version 2010, a computer-aided design program for mechanical modeling, was used to design the device. This allowed the different parts to be designed, then both the plans and other information necessary for the development of our device were extracted, taking into account certain parameters such as the injector or cone position, the direction of the injector or cone, the collector distance, and injector support or cone, as discussed by the authors of [23,24].

**Figure 3:**
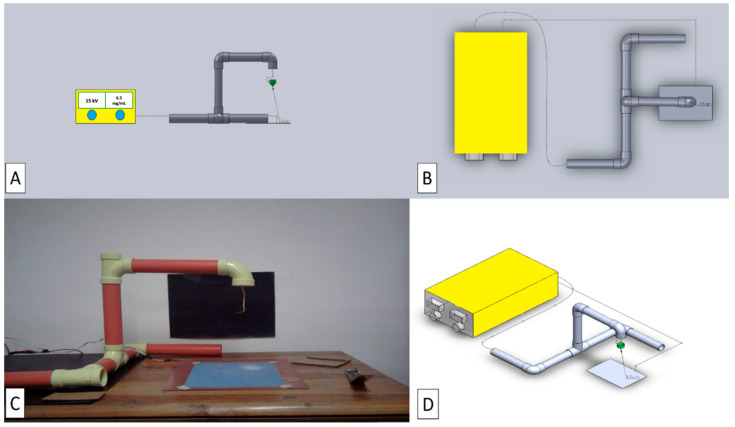
The basic configuration of a laboratory-made electrospinning device. (**A**,**B**,**D**) The Solidwork^®^ designs. (**C**) Macroscopic view of the electrospinning device.

The electrospinning technique was performed with a laboratory-made device, designed using Solidwork^®^ (2021 SP2.0, 175 Wyman Street Waltham, MA, USA) and constructed as shown in Figure 3. Therefore, it was decided to make the device with the injector or cone in the vertical position and direction, and a minimum distance of 15 cm or more was set. A Z-shaped support base was also used for better stability. Moreover, PVC tubes were chosen as the material used to direct the cables from the high-power source to the tip of the injector and to the collector inside them, to provide better security when carrying out the process of drawing out the fibers using the electrospinning technique.

In this project, the parameters that varied during the experiment were the voltage and the distance used to manufacture the polymeric fibers. According to the literature review, the voltage range in which nano and micrometric size fibers can be achieved by the electrospinning technique using polyethylene oxide is between 13 and 25 kV when dissolved in water. Conversely, the minimum distance limit between the polymer outlet cone and the collector is 10 cm, up to a maximum distance of 20 cm. Thus, the distances used in this experimental stage to obtain the fibers were 15, 16.5, and 19 cm.

Table 7, below, summarizes the experimental conditions used to manufacture the fibers using the electrospinning technique.

#### 2.4.4. Determination of the Diameter of the Fibers Using Scanning Electron Microscopy (SEM)

To analyze the diameter of the polyethylene oxide fibers, it was essential to have access to a field-emission microscope JEOL JSM 7600F (JEOL, 3-1-2 Musashino, Akishima, Tokyo, Japan), in which the samples were subjected to scanning utilizing an electron beam, which converts the electrical signals into textured-surface images that can be observed on the computer monitor. Since the samples were not conductive, they required prior preparation with a thin layer of gold. A small quantity of graphite tape was placed in a metal sample holder. A small portion of the sample to be analyzed was cut out and placed on the adhesive tape. Then, these samples were coated with a thin layer of metallic gold via plasma-assisted cathodic pulverization at a voltage of around 1 kV for 2 min. After this, the samples were placed in a tray, and electron microscope images were acquired from different areas of the samples at different amplification magnitudes. Porosity percentage and average fiber diameter were evaluated using the image analysis software ImageJ 8 (NIH, Bethesda, MD, USA), as documented in the study by Álvarez-Suárez et al. in 2017 [41].

## 3. Results

### 3.1. Model Testing with the Dataset

The model was tested with 10% of the dataset (10 records) that had not been used in either training or testing. The results showed an average error of 0.1362%, which implies that the model predicted the diameter of a fiber with an accuracy of 99.8638%. Table 4 shows the results of the predictive model with the testing dataset (Table 8).

The absolute difference (column *C*) is the absolute difference between the real and the predicted diameter:

C=ABS(A−B).

Furthermore, the relative error was calculated proportionally, between the absolute difference and the actual diameter:

$$D=\frac{C}{A}\times 100.$$

Finally, the relative success was calculated with the percentage complement of the relative error:

E=100−D.

**Figure 4:**
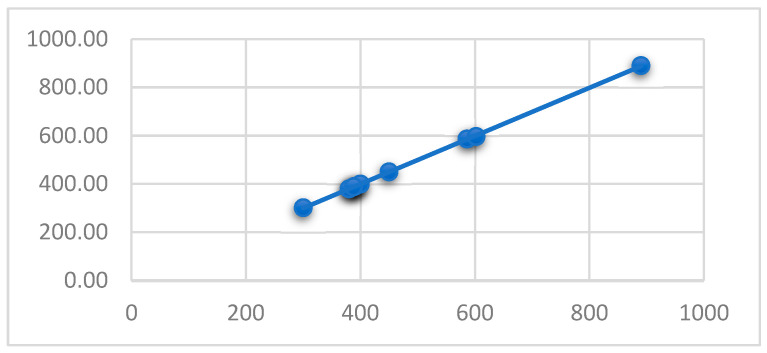
Correlation between the real and predicted dataset diameter values, using the testing dataset.

Additionally, Figure 4 shows the behavior of the resulting data concerning the actual data, which correlates to 0.99995, implying a practically 1-to-1 relationship between the real dataset and the data from the model results.

Thus, the prediction model’s test results indicate the feasibility of using the model to predict nanofiber diameter.

### 3.2. Model Validation with the Laboratory-Produced Fibers

In addition to traditional testing via dataset segmentation, in this work, a dataset was generated in the laboratory to evaluate the prediction model, as shown in Section 3.3. By introducing the same parameters into the prediction model, the results shown in Table 9 were obtained.

The relative error that the model threw out when evaluating the laboratory dataset was 2.2864%; consequently, the model’s prediction has an accuracy of 97.7136%.

**Figure 5:**
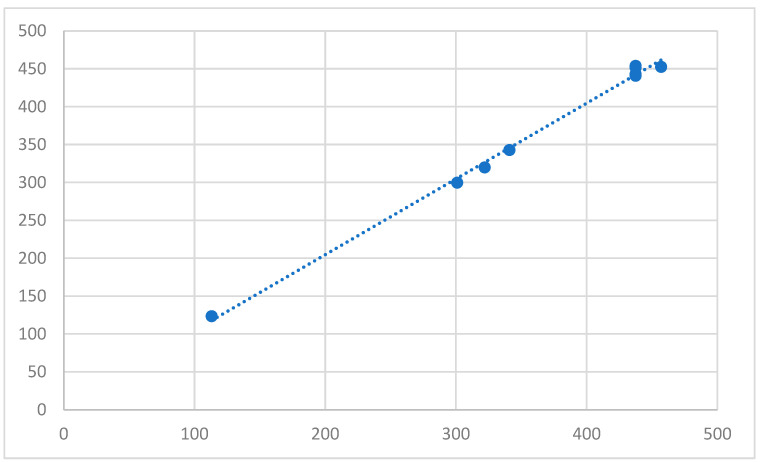
Correlation between the laboratory and the predicted dataset’s diameters, using the testing dataset.

Figure 5 also shows the correlation between the laboratory and the predicted dataset, which is 0.99786.

The results obtained on the laboratory dataset differ slightly from those produced by the model on the testing dataset. In Section 4, a discussion about the reasons and implications of this variation is presented.

### 3.3. Implementation of the Prediction Model

One of the main characteristics of a neural network is that despite the complexity of the algorithms used to obtain the resulting model, once the training is concluded satisfactorily, the model can be implemented in various ways using the functionalities offered by the appropriate programming languages, until the algorithms are implemented in a set of basic mathematical operations.

Regarding implementation using the MATLAB model, we developed a basic prototype, as follows.

**Figure 6:**
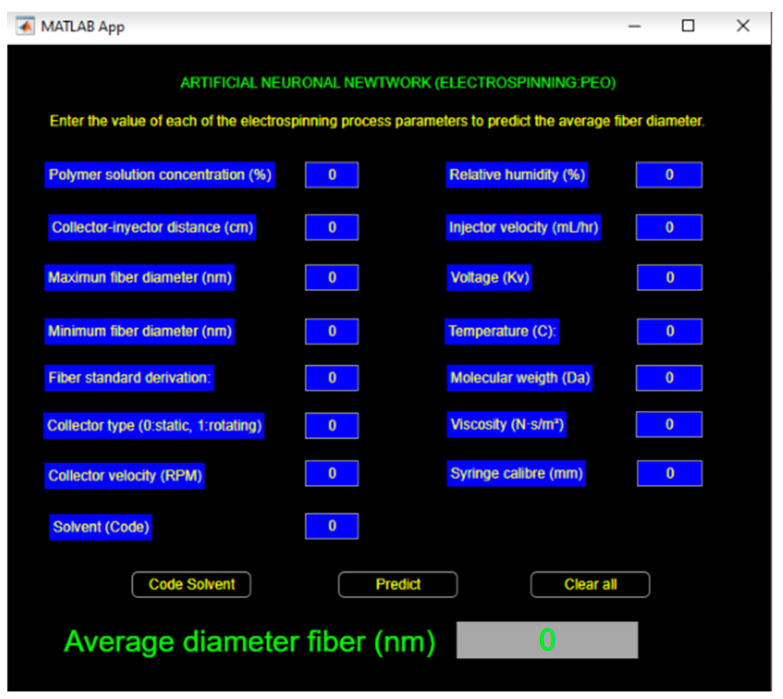
Prototype for the prediction model, using the MATLAB framework.

Since the definitive neural network for the prediction of the average fiber diameter was the one that included all the processing parameters and was designed with a layered architecture (15-15-1-1), we implemented a user interface for gathering those input parameters (see Figure 6), using the functionalities provided by the MATLAB framework.

**Figure 7:**
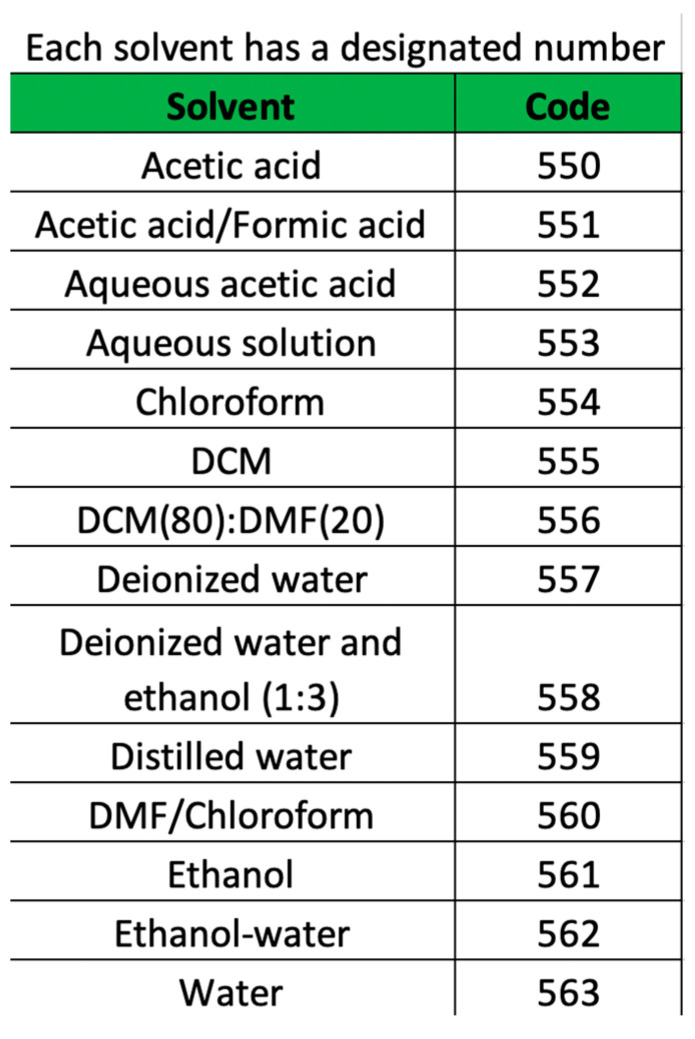
Solvent codes, as shown on the prototype model’s screen.

Because the “Solvent” parameter comprises categorical data, which implies that it must be numerically coded to use it as an input to the prediction model, this information is presented via the user interface (see Figure 7).

Furthermore, for the mathematical implementation, it is vital to know the weighting associated with the input variables. This is shown in Table 10, wherein each row corresponds to a neuron, and each column corresponds to the input variables, while at each intersection, the corresponding weight is indicated; for simplicity of presentation, these numbers have been rounded to two decimal places. The total precision weights table can be provided by the authors upon request.

Each of the neurons passes through the sigmoid function before moving to the next layer, via the following equation:

$$S\left(n_{i}\right)=\frac{1}{1+e^{n_{i}}}.$$

Subsequently, the next layer is calculated using the weights established in Table 11.

To finish, the results of these weights are processed using the RELU function, using the following equation:

$$R\left(n_{16}\right)=max\;(0,n_{16})\left\{\begin{array}{c} 0\;for\;n_{16}\leq 0\\ n_{16}\;for\;n_{16}>0 \end{array}\right..$$

In this way, it is possible to implement this model, even without the use of a MATLAB executable file.

## 4. Discussion

This paper presents a PEO-based nanofiber diameter prediction model that has been obtained from a neural network algorithm, which was evaluated both traditionally, by partitioning the dataset obtained from the literature [6,7,8,10,20,22,23,25,26,27,28,29,30,31,32,42,43,44], and by using a dataset generated in the laboratory.

The results show a slight variation between both evaluation approaches: using the dataset obtained from the literature, the results yield results with an average error of 0.1362%, which is an improvement upon previous works, while the correlation is 0.99995. With the laboratory data, the results show an average error of 2.2864%, with a correlation coefficient of 0.99786. This implies an absolute difference of 2.1502%, while a fundamental difference of 0.00209 is observed regarding the correlation coefficient.

A possible explanation for the differences in the results is that not all the values of the training dataset were complete; therefore, it was completed synthetically by employing neural networks and searching for approximate information. In the case of tests using 10% of the dataset, it is understandable that the match is more exact since the values of that dataset share behavior patterns with the training dataset; thus, the prediction trend will be much more compatible. However, it is probable that the behavior patterns demonstrated in other laboratories or experiments are not the same as those demonstrated in the laboratories or investigations that do report those parameters, which leads to that type of pattern not being “taught” to the model; therefore, it cannot be identified at the time of the prediction.

This implies that although the importance and utility of neural network models for the prediction of the diameter of nanofibers via electrospinning are evident, it is also necessary to expand the dataset for model training to have information on all the parameters. Above all, it is necessary to carry out tests with nanofibers that are not represented in the model, to measure the genuine accuracy of the model.

Comparing this study with the literature [18], our results are derived from a dataset and experiments that consider ambient parameters, feed rate, and the nozzle parameter; these are parameters that were excluded by the mentioned article, making our study more robust and complete. Moreover, that study uses different polymer systems to feed and train the ANN. In our research, we used only PEO electrospun systems, whereby this sole parameter incremented the relative error of the survey. In conclusion, both studies (Brooks and Tucker (2015) and this study) presented different results that will enrich the use of ANN to predict fiber diameter [18].

In the revised literature [18,43,44,45], studies that apply an ANN to the electrospinning technique use fewer parameters such as voltage, collector–injector distance, and polymer concentration to predict the fiber diameter. This work aims to evaluate the reduction of the percentage error obtained when an increase in the parameter data is used to feed the input dataset. This reduction was demonstrated in this study, and several parameters that were not considered previously were shown to affect the final result.

## 5. Conclusions

This paper proposes a model to predict the diameter of a PEO nanofiber produced by electrospinning, using 15 input parameters. The model was generated from a neural network that was trained with 105 parameters drawn from a literature review, using the Bayesian regulation method. Since the choice of input parameters in order to develop a nanofiber by electrospinning is an empirical procedure, which is carried out mainly by trial and error, its success depends greatly on the expertise of the person carrying out the process; when the input parameters are not correct, the nanofiber is not adequate for the application for which it was intended, which causes wastage of resources.

The results of this work show the relevance of using neural networks for the prediction of nanofiber diameter since the results show that the projections are strongly correlated to the real values. However, it is essential to note that when evaluating parameters with nanofibers that are not represented in the dataset, such as those we generated for evaluation, a slight decrease in the accuracy of the model appeared. Hence, the need to continue this research is evident; this can be achieved by increasing the dataset and the number of parameters used in the model, and by testing the prediction models with data that are utterly independent of the training dataset.

## Figures and Tables

**Table 1:** Some of the most critical parameters to determine the fiber diameter average (according to [14]).

Parameters of the Solution	Parameters of the Process	Ambiental Parameters
The molecular weight of the polymer	Applied voltage	Temperature
Higher molecular weight leads to a lower probability of generating drops on the collector	Higher voltage provokes higher drip probability	Higher temperature permits a lower fiber diameter
Polymer concentration	Collector distance	Humidity
Higher concentration provokes fewer drips and thicker fibers	Greater distance improves lower fiber diameter and causes less humidity	Low humidity permits rapid solvent evaporation
Solution conductivity	Flow-rate	
Increased conductivity reduces defects in the fibers and reduces their diameters	A higher rate produces higher humidity of the fibers	
Surface tension	Collector	
Lower tension promotes fiber formation	Different collectors produce different fiber patterns and distribution	

**Table 2:** Codes and weighting for the solvents.

Code	Normalized Code	Solvent	Dielectric Constant	Dipole Moment
550	0	Acetic acid	6.2, 20 °C	3.3
551	0.077	Acetic Acid/Formic acid	51.1, 25 °C	1.74
552	0.153	Aqueous acetic acid	6.2, 20 °C	1.74
553	0.230	Aqueous solution	78.3	1.85
554	0.307	Chloroform	4.8, 20 °C	1.15
555	0.385	DCM	8.93, 25 °C	1.14
556	0.461	DCM (80): DMF (20)	8.93, 25 °C:37.51, 25 °C	1.14:3.86
557	0.538	Deionized water	78.57, 25 °C	1.86
558	0.615	Deionized water and ethanol (1:3)	77.82,22 °C	3.1
559	0.692	Distilled water	78.57, 25 °C	1.8
560	0.769	DMF/Chloroform	37.51, 25 °C: 4.8, 20 °C	1.14:1.15
561	0.846	Ethanol	33, 20 °C	2.04
562	0.923	Ethanol–water	80.1, 25.3 °C	3.1

DCM: Dichloromethane; DMF: Dimethyl formamide.

**Table 3:** Available values for each parameter in the dataset.

Parameter	Available Values
Molecular weight (Da)	12
Solvent	15
Polymer solution concentration (%)	18
Voltage (kV)	15
Collector-injector distance (cm)	10
Injector velocity (flow rate (mL/h))	7
Collector type	2
Collector velocity (rpm)	18
Relative humidity (%)	12
Temperature (°C)	11
Fiber standard deviation	71
Minimum fiber diameter	96
Maximum fiber diameter	98
Viscosity (N·s/m^2^)	65
Syringe caliber (mm)	3

**Table 4:** Evaluation of the relevance of the input variables.

Iteration	Parameters	% Correlation
1	1, 3, 4, 7, 9, 13, 14 and 15	0.99993
2	Iteration 1 + parameter 8	1
3	Iteration 2 + parameters 11 and 12	0.99998
4	Iteration 3 + parameters 5 and 6	0.99999
5	Iteration 4 + parameter 10	0.99999
6	Iteration 5 + parameter 2	0.99999

Parameters: (1) Molecular weight; (2) solvent; (3) polymer solution concentration; (4) voltage; (5) viscosity; (6) syringe caliber; (7) injector velocity (flow rate); (8) collector type; (9) collector–injector distance; (10) collector velocity; (11) relative humidity; (12) temperature; (13) fiber standard deviation; (14) minimum fiber diameter; (15) maximum fiber diameter.

**Table 5:** Comparison between the percentage errors of the dataset versus real cases.

No.	Parameter	Dataset	Real Cases
1	Molecular weight	1.1175	7.06
2	Solvent	0.1007	9.45
3	Polymer solution concentration	0.1456	6.35
4	Voltage	0.3151	3.12
5	Viscosity	0.7037	5.01
6	Syringe caliber	0.2636	3
7	Injector velocity (flow rate)	0.2021	3.05
8	Collector type	0.0726	12.04
9	Collector–injector distance	1.6834	3.18
10	Collector velocity	2.0437	16.46
11	Relative humidity	0.5533	4.99
12	Temperature	0.0515	6.68
13	Fiber standard deviation	0.7604	30.32
14	Minimum fiber diameter	0.0574	3.33
15	Maximum fiber diameter	0.057	3.75
	Average % error	0.5348	7.85

**Table 6:** Hyperparameters established for tuning.

Iteration	Layers	Activation Function	Epochs	Optimization Algorithm	MSE	% Correlation
1	8-8-1-1	Sigmoid-Relu	1000	Bayesian Regularization	2.35	0.99993
2	9-9-1-1	Sigmoid-Relu	1000	Bayesian Regularization	7.43	1
3	11-11-1-1	Sigmoid-Relu	1000	Bayesian Regularization	12.5	0.99998
4	13-13-1-1	Sigmoid-Relu	1000	Bayesian Regularization	4.58	0.99999
5	14-14-1-1	Sigmoid-Relu	1000	Bayesian Regularization	1.12	0.99999
6	15-15-1-1	Sigmoid-Relu	1000	Bayesian Regularization	8.06	0.99999

**Table 7:** Combinations of voltage vs. distance for manufacturing polymeric fibers (constant parameters: 6.5% PEO; time—1 h).

No.	Nomenclature	Distance (cm)	Voltage (kV)
1	PEO19D15V	19	15
2	PEO19D16.5V	19	16.5
3	PEO19D18V	19	18
4	PEO17D15V	17	15
5	PEO17D16.5V	17	16.5
6	PEO17D18V	17	18
7	PEO15D15V	15	15
8	PEO15V17.6V	15	16.5
9	PEO15D18V	15	18

**Table 8:** Results with the testing dataset.

Real Diameter (A)	Predicted Diameter (B)	Absolute Difference (C)	Relative Error (D)	Relative Success (E)
450	450.00	0.00	0.0006	99.9994
390	390.00	0.00	0.0002	99.9998
890	890.00	0.00	0.0000	100.0000
586	586.00	0.00	0.0000	100.0000
602	596.75	5.25	0.8726	99.1274
300	301.39	1.39	0.4620	99.5380
390	390.01	0.01	0.0036	99.9964
380	379.96	0.04	0.0104	99.9896
400	399.99	0.01	0.0031	99.9969
388	388.04	0.04	0.0099	99.9901

**Table 9:** Results on the laboratory testing dataset.

No.	Nomenclature	Real Diameter (A)	Predicted Diameter (B)	Absolute Difference (C)	Relative Error (D)	Relative Success (E)
1	PEO19D15V	437.5	453.7	16.2	3.7029	96.2971
2	PEO19D16.5V	437.5	451.4	13.9	3.1771	96.8229
3	PEO19D18V	113.15	123.4	10.25	9.0588	90.9412
4	PEO17D15V	437.5	443	5.5	1.2571	98.7429
5	PEO17D16.5V	437.5	440.8	3.3	0.7543	99.2457
6	PEO17D18V	341	342.8	1.8	0.5279	99.4721
7	PEO15D15V	301	299.7	1.3	0.4319	99.5681
8	PEO15V17.6V	322	319.8	2.2	0.6832	99.3168
9	PEO15D18V	457	452.5	4.5	0.9847	99.0153

**Table 10:** Hidden layer weights.

n	i1	i2	i3	i4	i5	i6	i7	i8	i9	i10	i11	i12	i13	i14	i15	Bias
1	−0.25	0.01	−0.12	−0.16	−0.04	0.05	0.14	0.13	−0.001	−0.13	−0.03	−0.02	0.46	−0.1	0.09	0.07
2	−0.05	0.11	−0.03	0.007	−0.24	0.04	0.03	0.06	−0.03	−0.04	0.21	−0.07	−0.34	−0.06	−0.01	0.04
3	0.01	0.004	0.03	0.004	−0.001	0.003	−0.01	0.04	−0.002	−0.01	0.002	−0.01	0.01	0.004	0.004	−0.002
4	−0.02	−0.01	−0.03	0.001	0.01	0.001	0.05	−0.06	0.01	0.01	−0.02	0.06	−0.22	0.002	−0.004	−0.01
5	−0.15	−0.01	0.03	−0.11	0.01	0.01	−0.17	−0.13	0.10	0.12	0.03	0.02	−0.31	0.19	−0.03	−0.01
6	0.01	0.01	0.03	0.003	−0.01	−0.004	−0.05	0.05	−0.002	0.01	0.01	−0.06	0.23	−0.01	−0.002	0.02
7	−0.02	0.06	0.08	0.11	0.02	−0.06	−0.10	0.04	−0.01	−0.16	0.18	−0.11	−0.34	−0.09	0.08	−0.01
8	−0.01	−0.001	0.03	−0.01	−0.02	−0.02	−0.07	0.05	−0.01	−0.003	−0.02	−0.06	0.26	−0.02	−0.02	0.02
9	−0.03	−0.02	−0.02	−0.01	0.01	−0.01	0.05	−0.03	0.002	0.02	0.01	0.04	−0.23	0.01	0.01	−0.02
10	0.14	−0.11	−0.06	−0.04	0.03	0.08	−0.05	−0.01	−0.08	−0.04	0.02	−0.003	−0.26	−0.39	−0.07	−0.06
11	0.01	0.04	−0.08	0.01	0.10	0.002	0.14	0.06	0.04	−0.10	0.21	0.03	−0.28	0.08	0.01	−0.02
12	−0.12	−0.06	0.05	−0.16	−0.01	−0.01	0.01	0.08	−0.03	0.03	0.11	−0.07	−0.35	0.08	0.12	−0.07
13	−0.01	−0.002	−0.04	0.002	0.01	0.01	0.05	−0.06	0.004	0.008	−0.03	0.06	−0.21	0.001	−0.01	−0.01
14	−0.01	−0.001	−0.04	0.002	0.01	0.01	0.05	−0.06	0.003	0.008	−0.02	0.06	−0.22	0.004	−0.004	−0.01
15	0.004	0.003	0.02	0.003	0.0001	0.004	−0.01	0.02	−0.002	−0.001	−0.002	0.004	0.04	0.003	0.001	−0.01

**Table 11:** Outcome layer weights.

n	sn1	sn2	sn3	sn4	sn5	sn6	sn7	sn8	sn9	sn10	sn11	sn12	sn13	sn14	sn15	Bias
16	0.49	−0.42	0.12	−0.26	−0.44	0.27	−0.43	0.30	−0.26	−0.45	−0.38	−0.41	−0.25	−0.26	0.05	−0.10

## Data Availability

The training dataset and the prediction model will be shared upon request to the authors.

## Supplementary Materials

The following supporting information can be downloaded at: https://www.mdpi.com/article/10.3390/mi14071410/s1. Table S1: Full precision ANN weights; Video S1: The model fitting process.
